# Letter from Dr. Q. C. Smith, Including Letter of Dr. Maclean

**Published:** 1896-09

**Authors:** 


### Correspondence.

AUSTIN, TEXAS, August 5, 1896.

*Editors Texas Medical Journal:*

The JOURNAL for August came duly, and was perused with interest. Please republish Prof. Donald Maclean's letter, as it appears in the *Journal of the American Medical Association* for July 25, 1896, as germane to the matter now under consideration:

"The Practice of Medicine according to Law." 'Tis astonishing to any intelligent M. D. that so few physicians seem to comprehend the marked difference between *clinical* and *State medicine.* The latter, as its name clearly implies, is very properly a most important function of the State. The former is, or *should be*, the most capable application of *curative therapeutics*, that available knowledge will permit. And the methods of said application, or means used, should be left utterly untrammeled by any legislative or State enactments whatever. Or, as the great Bowling said: "Touch it *not* with the *dirty* finger of the *law.*"

And as we published, many years ago, the history of civilization, as recorded by Buckle and Draper, plainly shows, that in every age of the world in which the State dominated in the application of scientific knowledge, that progress was thereby seriously retarded, hampered and perverted, and the tree of true and useful knowledge dwarfed and withered.

The remedy is at hand. Let every *legal quack* become and *act* equal to his *pretensions;* then so-called quacks will starve in "innocuous disuetude," and the millennium in medicine surely dawn.

Yours fraternally,

Q. C. SMITH, M. D.

617 Colorado Street.

AN OPEN LETTER TO THE MEMBERS AND FRIENDS OF THE MEDICAL PROFESSION (REGULAR) IN MICHIGAN.

DETROIT, MICH., July 20, 1896.

An organization calling itself "The Michigan Medical Legislative League" has, in a printed circular, appealed to you and to me for aid and comfort in its efforts to accomplish a certain self-imposed task, namely: "To secure through organized effort just and equitable laws regulating the practice of medicine in

Michigan and to promote the interests of the medical fraternity." Truly a noble object and worthy the utmost effort of every lover of science and friend of humanity. The Executive Board of the League consists of three irregulars and two members of the regular profession.

For almost a quarter of a century, in this State, I and my professional associates have labored and waited and hoped for the accomplishment of this, which is plainly declared to be the main "object" of the "Legislative League." It is needless to say that its attainment is as dear to our hearts to-day as it ever was. We stand ready now as in times past to do anything and everything within the bounds of honor and decency to secure legal medical protection for the sick and suffering. It is therefore with extreme sorrow that we feel compelled to say that the course of procedure and policy publicly avowed by the "Legislative League" is, in our opinion, deserving only the pity and contempt of every true friend of regular medicine and every individual who sincerely wishes to do what he can for the relief and protection of the sick and hurt. The plainly avowed ultimate "object" of the so-called league is the securing of an act of the legislature, as follows:

A bill to establish a Board of Registration and to regulate the practice of medicine, etc. Of this proposed bill Section 1 reads: "The people of the State of Michigan enact that the governor shall appoint nine physicians, residents of the State, *not more than four of whom shall be regular*, two homeopathic, two eclectic, one physiomedical (whatever that may be), who shall constitute a Board of Registration of Medicine."

Section 3 reads: "All persons engaged in the practice of medicine and surgery in any of its branches, and all who wish to begin the same in the State, shall apply to this Board to be registered, and for a certificate of such registration."

If this bill had originated with non-professional politicians, or with quacks and irregulars, the pitiful degree of recognition accorded the adherents of true science and the followers of every great and noble name in medicine and surgery from Hippocrates to Pasteur and Lister might have been dismissed with a smile of ridicule and contempt; but coming, as it appears to come, from men who still profess loyalty to honest scientific medicine, words fail us to adequately express our amazement and sense of humiliation. The regular profession of Michigan numerically exceed all other so-called schools or sects in the proportion of two to one. In the matter of talent, education, character and influence the difference is surely immensely greater. Can it then be possible that the representatives of the great profession of honest scientific medicine in this State are ready and willing to join forces with every quack and pathy and sect for the purpose of securing a law which, in the most practical and unmistakable manner declares the inferiority of regular and the superiority of irregular medicine? Many years ago the profession of this State submitted to a severe rebuke at the hands of the American Medical Association for having even thought of a somewhat similar proposition. Can it be possible that at this late date the regular profession in Michigan feels itself so feeble and unable to maintain itself that it stands ready to defy the public professional sentiment of this and all other lands, and in the hope of obtaining a little milk and water degree of protection (*for themselves rather than their patients*) enter into a combination and unholy alliance with its most insidious and meanest foes, and place the balance of power in the hands of the enemy?

The occasion is, in our opinion, critical. The honor and the good name of the profession in Michigan is at stake. It is not yet too late to rescue that good name and keep it safe from disgrace and misfortune. For myself and others who love and honor our profession, we earnestly implore the members to come forward at this time and openly approve the only attitude consistent with the honor and best interests of the profession. Let us earnestly and persistently oppose any and all legislation which tends to increase the power of the quack, the charlatan and the sectarian, and which at the same time degrades and humiliates the only real school of medicine, namely, that school which rises above all pathies and all sects and seeks only the welfare of the sick and suffering, and in doing so eagerly accepts anything and everything which holds out reasonable hope of being practically useful. Let us as a profession demonstrate by our lives and our works our claims to legislative recognition and convince the people that *our* interests are *their* interests.

Legislation so earned and secured would be a glorious triumph for science and for humanity. Legislation obtained by compromise and abdication and unholy alliance with everything and every creature in the shape of a medical parasite only confers upon the latter undeserved honor, while it stamps the word *shame* upon the brow of each one of us in Michigan who claims to be, to the best of his ability, the representative and the exponent of the noblest of all professions.

Perfection cannot be claimed for any man or set of men, and we have no choice but to confess to many failures and imperfections on the part of the regular profession in this State and anywhere else. Nevertheless, the fact remains that our aims are avowedly higher and in practice our record incomparably grander than that of all the other so-called schools and sects combined.

Why, therefore, in the name of all that is true and good, should we condescend to join hands with any sect, school or pathy, thereby uplifting that which we condemn and despise, and at the same time dishonoring that for which we have always been willing to do our utmost to protect and save?

DONALD MACLEAN, M. D.

